# TIMAP, a Regulatory Subunit of Protein Phosphatase 1, Inhibits In Vitro Neuronal Differentiation

**DOI:** 10.3390/ijms242417360

**Published:** 2023-12-11

**Authors:** Márton Fonódi, Zsófia Thalwieser, Csilla Csortos, Anita Boratkó

**Affiliations:** Department of Medical Chemistry, Faculty of Medicine, University of Debrecen, Egyetem Tér 1, H-4032 Debrecen, Hungary; fonodi.marton@med.unideb.hu (M.F.); thalwieser.zsofia@med.unideb.hu (Z.T.); csortos@gmail.com (C.C.)

**Keywords:** TIMAP, protein phosphatase, neuroblastoma, differentiation, interactome

## Abstract

TIMAP (TGF-β-inhibited membrane associated protein) is abundant in endothelial cells, and it has been regarded as a member of the myosin phosphatase targeting protein (MYPT) family. Our workgroup previously identified several interacting protein partners of TIMAP and proved its regulatory subunit role for protein phosphatase 1 catalytic subunit (PP1c). TIMAP is also expressed in neuronal cells, but details of its function have not been studied yet. Therefore, we aimed to explore the role of TIMAP in neuronal cells, especially during differentiation. Expression of TIMAP was proved both at mRNA and protein levels in SH-SY5Y human neuroblastoma cells. Differentiation of SH-SY5Y cells was optimized and proved by the detection of neuronal differentiation markers, such as β3-tubulin, nestin and inhibitor of differentiation 1 (ID1) using qPCR and Western blot. We found downregulation of TIMAP during differentiation. In accordance with this, overexpression of recombinant TIMAP attenuated the differentiation of neuronal cells. Moreover, the subcellular localization of TIMAP has changed during differentiation as it translocated from the plasma membrane into the nucleus. The nuclear interactome of TIMAP revealed more than 50 proteins, offering the possibility to further investigate the role of TIMAP in several key physiological pathways of neuronal cells.

## 1. Introduction

Reversible protein phosphorylation is a fundamental and crucial posttranslational modification of proteins that regulates the activity, conformation, interacting partners or cellular fate of proteins. Protein kinases attach a phosphate group to Ser, Thr or Tyr side chain of proteins [1], while protein phosphatases are able to remove it by hydrolysis [2,3]. Protein phosphatase 1 (PP1) enzyme was the first identified Ser/Thr phosphatase [2,4], and it is responsible for the dephosphorylation of over half of the phosphoserine/threonine residues in eukaryotic cells [5]. It takes part in many intracellular processes in eukaryotic cells, such as protein translation, apoptosis, glycogen metabolism, signal transduction and RNA processing [2,6]. Structurally active PP1 holoenzymes consist of a catalytic subunit in complex with at least one regulatory subunit, also known as PP1 interacting protein (PIP). The ~37 kDa catalytic subunits, PP1c, are highly preserved among the species and are encoded by three genes in mammals: *PPP1CA*, *PPP1CB* and *PPP1CC*, resulting in four isoforms PP1cα, PP1cβ (a.k.a. PP1cδ), PP1cγ1 and PP1cγ2 (resulting from alternative splicing) [2,7,8,9,10,11]. The expression of all isoforms is widespread except for PP1γ2, which is predominantly found in testes and sperm [8].

TIMAP (TGF-β-inhibited membrane-associated protein) belongs to the myosin phosphatase targeting protein (MYPT) family as one of the regulatory subunits of the PP1 holoenzyme [12]. TIMAP is predominant in hematopoietic cells, and an increasing number of papers explore its function in endothelial cells [13]. As a PP1 regulatory subunit, its main function is considered to control the activity of the δ isoform of PP1c [14,15]. TIMAP undergoes reversible phosphorylation on multiple Ser side chains, and the substrate specificity of TIMAP–PP1c relies on its phosphorylation state [12,16]. Among other phosphoproteins, ezrin–radixin–moesin (ERM) and merlin (neurofibromin 2; NF2) were identified as TIMAP–PP1c substrates in endothelial cells (reviewed in [13]). ERM proteins and merlin have a prominent role in neuronal cell functions, and their altered expression or posttranslational regulation are associated with serious diseases, for example, neurofibromatosis [17]. TIMAP mRNA was detected in tissues derived from the rat central nervous system such as the cerebellum, forebrain or brainstem; however, no detailed research was carried out in neuronal cells [14,18]. The coding gene (*ppp1r16b*) for TIMAP was identified as a prognostic biomarker of glioblastoma multiforme, a type of primary brain tumor, shown by integrated analysis of methylation and expression profiles [19]. The high expression level and the limited knowledge about TIMAP in neuronal cells raised our interest in studying the role of TIMAP in these cell types.

SH-SY5Y human neuroblastoma cell line is widely utilized as an in vitro model system in neurobiology, in particular for research focusing on neurodegenerative diseases such as Alzheimer’s disease [20], Parkinson’s disease [21]; neuronal aging [22], neuronal toxicology [23] as well as in the field of neurovirology [24]. The most advantageous quality of the SH-SY5Y cell line lies in its capability of being able to be differentiated into neuron-like phenotype exhibiting morphological and gene expressional similarities to that of mature neurons [25]. Undifferentiated SH-SY5Y cells show rounded morphology, non-polarized cell bodies with a low number of truncated cell processes [26]. When SH-SY5Y cells are fully differentiated into a neuron-like phenotype, they exhibit extended, often branched processes as well as withdrawal from the cell cycle [27]. Beside the morphological alterations, gene up- or downregulation are both easy-to-follow hallmarks of the differentiation. SH-SY5Y cells express neuronal markers such as proliferating cell nuclear antigen (PCNA), nestin, limited amount of dopamine-ß-hydroxylase (DBH), neurofilament proteins as well as nerve growth factor receptors (NGFR, butyryl-cholinesterase (BChE), acetylcholinesterase (AChE), tyrosine hydroxylase (TH) and basal noradrenaline release [25,26,27]. Differentiated cells express a variety of mature neuronal markers including growth-associated protein (GAP-43), synaptophysin (SYN), synaptic vesicle protein II (SV2), neuronal nuclei antigen (NeuN), neuron-specific enolase (NSE) and microtubule-associated protein-2 (MAP-2), β3-tubulin or high-weight neurofilament (NF-H) [25]. These features of SH-SY5Y were utilized in the present study to explore the role of TIMAP in neuronal cells, especially in differentiation.

## 2. Results

### 2.1. TIMAP–PP1c Complex Is Present in SH-SY5Y Cells

**Figure 1:**
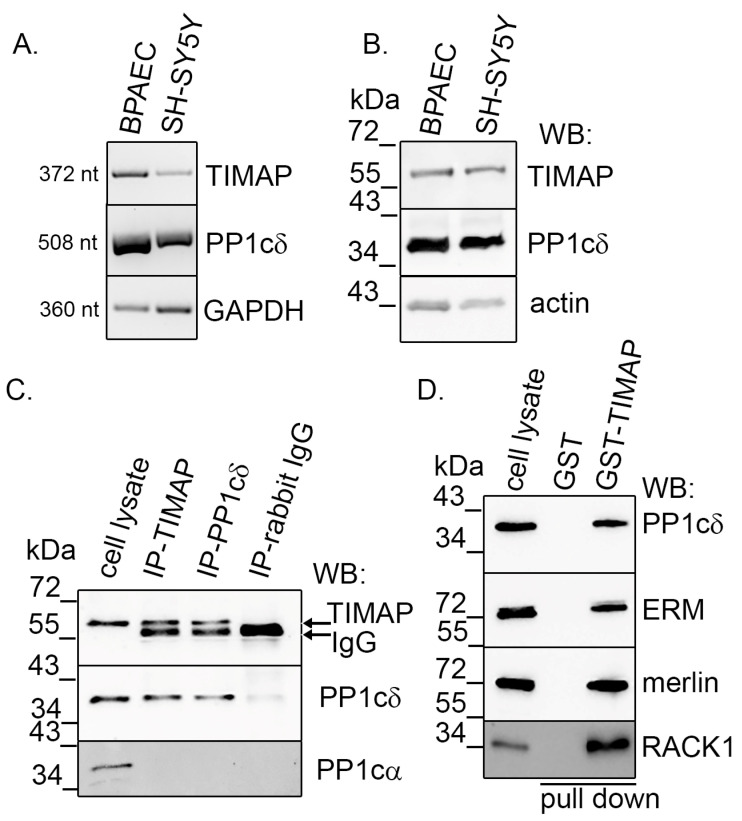
Detection of TIMAP–PP1cδ in SH-SY5Y cells. (**A**) RT-PCR was performed using specific primer pairs that targeted TIMAP, PP1cδ and GAPDH in BPAEC and SH-SY5Y cells. (**B**) Western blot analysis of BPAEC and SH-SY5Y cells using TIMAP- and PP1cδ-specific antibodies. Actin was utilized as a loading control. (**C**) TIMAP or PP1cδ was subjected to immunoprecipitation from lysates of SH-SY5Y cells. IP complexes were subjected to Western blot to detect the presence of TIMAP, PP1cδ and PP1cα. The negative control utilized was rabbit IgG. (**D**) Glutathione S transferase (GST) and GST-TIMAP were immobilized on glutathione Sepharose 4B beads, then the resin samples were incubated with SH-SY5Y cell lysate. Samples were tested for TIMAP, ERM, merlin and RACK1 by Western blot.

SH-SY5Y human neuroblastoma cell line was used as a model system to study the TIMAP–PP1c complex in neuronal cells. First, we confirmed that TIMAP (*PPP1R16B*; UNIPROT: Q96T49) and PP1cδ (*PPP1CB*; UNIPROT: P62140) mRNA and proteins are present and expressed in these cells. RT-PCR was performed using total RNA isolated from SH-SY5Y cells. As a positive control, bovine pulmonary artery endothelial cell (BPAEC) cDNA was used, as TIMAP is highly expressed in endothelial cells [14]. Primers were designed to recognize both human and bovine sequences. PCR products were identified at the expected base pair sizes both with TIMAP- and PP1cδ-specific primer pairs (Figure 1A). The expressions of these genes at protein level were verified by Western blots from total SH-SY5Y and BPAEC cell lysate, as positive control. The utilized antibodies demonstrated the ability to detect both human and bovine proteins, as expected from the manufacturers. Figure 1B shows that SH-SY5Y cells express both TIMAP and PP1cδ. TIMAP is a well-known regulatory subunit of PP1cδ in endothelial cells, and several substrates of the active holoenzyme have also been described [13]. To confirm that TIMAP specifically binds to PP1cδ in neuronal cells, endogenous TIMAP–PP1cδ interaction was confirmed by immunoprecipitation using specific antibodies. TIMAP was detected in the immunoprecipitate of PP1cδ and vice versa; moreover, no interaction was detected with PP1cα (Figure 1C). GST-tagged bacterially expressed TIMAP protein was used in pull-down method to examine the binding of TIMAP–PP1c interacting partners/substrates described earlier in endothelial cells. Our results prove that ERM, merlin and receptor for activated C kinase 1 (RACK1) proteins bind to the TIMAP–PP1cδ complex in neuronal cells as well (Figure 1D).

### 2.2. TIMAP Is Downregulated in Differentiated SH-SY5Y Cells

**Figure 2:**
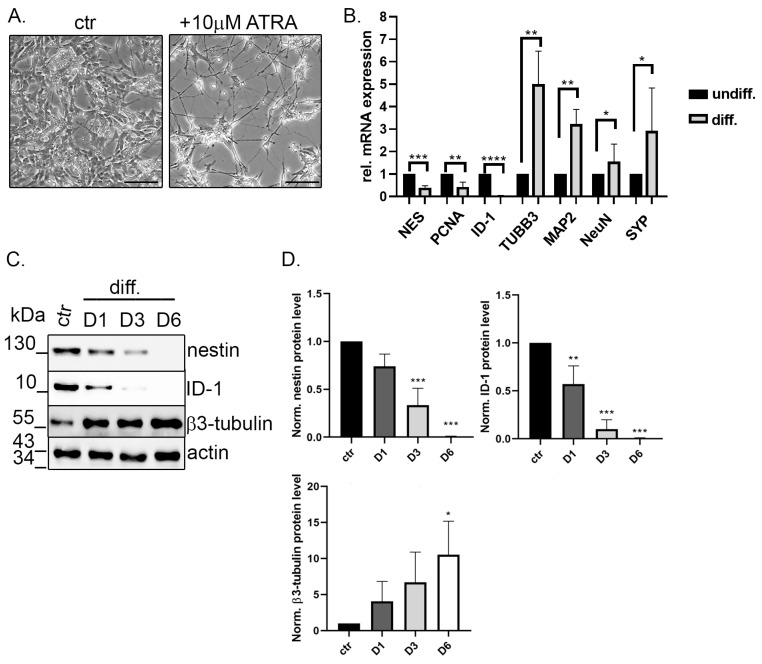
ATRA-mediated differentiation of SH-SY5Y cells. (**A**) SH-SY5Y cells were cultured with vehicle (DMSO, control) or ATRA for 6 days. Morphological changes related to differentiation were obtained using a Leica Axioscope microscope. Scale bar: 100 µm. (**B**) Cells were treated with vehicle (DMSO) (undiff) or ATRA for 6 days (diff). The relative mRNA expression of differentiation markers was assessed using qPCR analysis. Data are presented as mean ± SD (*n* = 3). Statistical differences were examined by unpaired *t*-test (* *p* < 0.05; ** *p* < 0.01, *** *p* < 0.001, **** *p* < 0.0001). (**C**) Western blot analysis of neuronal markers of untreated (ctr) or ATRA-treated cells collected on Day 1 (D1), Day 3 (D3) or Day 6 (D6). Actin was used as loading control. (**D**) The relative amount of nestin, ID-1 and β3-tubulin was determined by densitometry of Western blot results. Protein level normalization was performed using actin bands. Data are presented as mean ± SD (*n* = 3). Statistical analysis was performed with one-way ANOVA, Dunnett’s multiple comparisons test (* *p* < 0.05; ** *p* < 0.01, *** *p* < 0.001).

Next, differentiation of SH-SY5Y cells initiated by all trans retinoic acid (ATRA) was tested. Treated cells achieved a typical neuronal phenotype with extended neurites by the end of the 6th day, while the undifferentiated cells maintained relatively short neurites (Figure 2A). Relative levels of genes affected by differentiation were measured by qPCR. Undifferentiated and differentiated (Day 6 of ATRA treatment) cells were used to extract total RNA and qPCR was performed (Figure 2B). Unmature neuron markers like nestin (NES), inhibitor of differentiation 1 (ID-1) or proliferating cell nuclear antigen (PCNA) had a significantly lower level in differentiated cells, while mature neuronal markers like β3-tubulin (TUBB3), microtubule-associated protein 2 (MAP2), neuronal nuclei (NeuN) or synaptophysin (SYP) increased greatly, proving the successful differentiation of the cells (Figure 2B). Protein levels of ID-1, nestin and β3-tubulin were also compared by Western blot analysis during the differentiation by taking samples on Day 1, Day 3 and Day 6 (Figure 2C). A gradually decreasing protein expression level was found for ID-1 and nestin, in contrast with the intensifying β3-tubulin level. As the sign of full differentiation, both ID-1 and nestin proteins were undetectable at Day 6 (Figure 2C,D).

**Figure 3:**
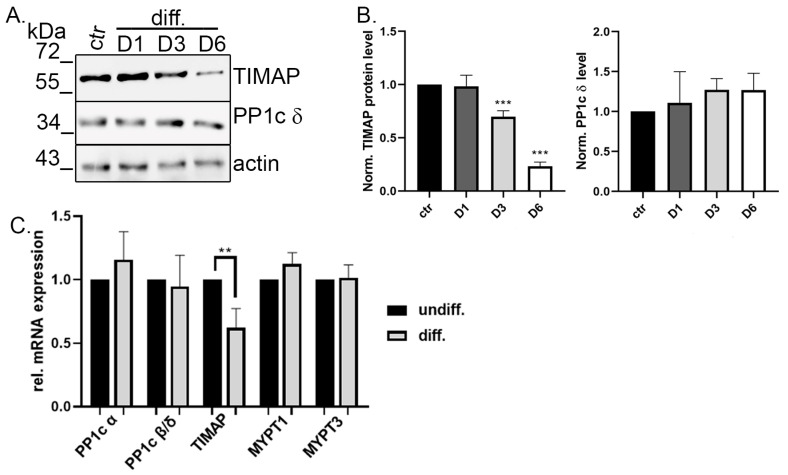
Expression level of TIMAP decreases during differentiation. (**A**) Western blot analysis of untreated (ctr) or ATRA-treated cells collected on Day 1 (D1), Day 3 (D3) or Day 6 (D6). Samples were tested using antibodies specific for TIMAP and PP1cδ. (**B**) The relative amount of TIMAP and PP1c was determined using densitometry of Western blot results, with the utilization of actin bands for protein level normalization. The results are presented as mean ± SD (*n* = 3). Statistical analysis was performed with one-way ANOVA, Dunnett’s multiple comparisons test (*** p < 0.001). (**C**) Cells were treated with vehicle (DMSO) or ATRA for 6 days. qPCR analysis shows the relative mRNA expression of PP1 catalytic (PP1cα; PP1cδ) or regulatory subunits (TIMAP, MYPT1, MYPT3). Data are given as mean ± SD (*n* = 4). Statistical analysis was performed using unpaired *t*-tests (** *p* < 0.01).

Then, we analyzed TIMAP and PP1cδ mRNA and protein expression levels during the differentiation. Protein samples, collected from control and Day 1, Day 3 and Day 6 of differentiation, were subjected to Western blot using TIMAP- and PP1cδ-specific antibodies (Figure 3A). The normalized protein level of PP1cδ did not show any changes, while the TIMAP protein level was significantly reduced on Day 6 compared to control (Day 0) (Figure 3B). qPCR results revealed that the relative mRNA expression level of TIMAP was also lower in differentiated cells, while no significant changes were detected for PP1cδ, or the other abundant catalytic subunit isoform, PP1cα. Other PP1 regulatory members of the MYPT family, namely myosin phosphatase target subunit 1 (MYPT1) or myosin phosphatase target subunit 3 (MYPT3), were also checked; however, no alterations were found (Figure 3C).

### 2.3. TIMAP Overexpression Delays Neuronal Differentiation

**Figure 4:**
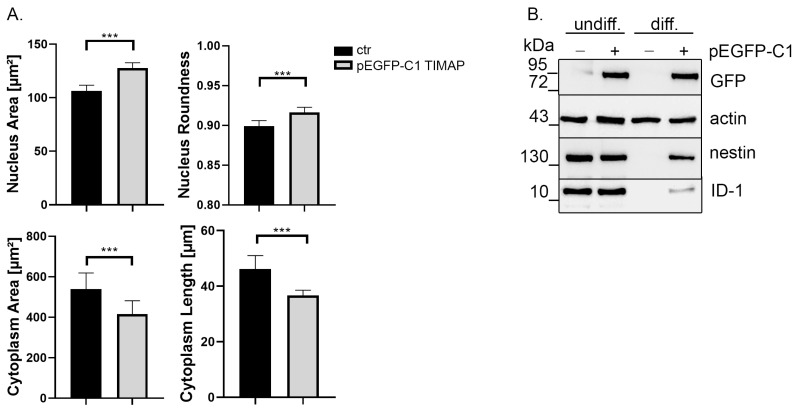
Overexpression of TIMAP attenuates differentiation. (**A**) Differentiated (Day 6) control and pEGFP-C1 TIMAP-expressing cells were analyzed using Opera Phenix HCS. Data are presented as mean ± SD (number of cells analyzed > 1000, from *n* = 3 experiments). Statistical analysis was performed using unpaired *t*-test (*** *p* < 0.001). (**B**) Control or pEGFP-C1 TIMAP-expressing cells were treated with vehicle (DMSO) (undiff) or ATRA (diff) for 6 days. Samples were tested using GFP-, nestin-, ID-1- and actin (loading control)-specific antibodies.

Subsequently, we tested the effect of TIMAP overexpression on differentiation and cell morphological changes of ATRA-treated SH-SY5Y cells. Cells were transiently transfected with pEGFP-C1 TIMAP plasmid. Transfected and non-transfected cells were tested for TIMAP antibody as well (Figure S1A). Differentiation 24 h post-transfection was performed as earlier. Morphological alterations were analyzed using Opera Phenix HCS (Figure S1B). Nucleus area and roundness were enlarged in transfected and differentiated cells, while cytoplasm area and length were significantly smaller compared to the differentiated cells without transfection (Figure 4A). This morphological phenotype of the TIMAP-overexpressing cells is similar to the undifferentiated cell phenotype, suggesting that overexpression of TIMAP attenuates neuronal differentiation. Therefore, differentiation markers were tested at protein level (Figure 4B). Untransfected and TIMAP transfected cells were differentiated, and samples were collected on Day 6. The strong GFP signal of the transfected cell sample shows that the recombinant TIMAP was present during the differentiation. In the untransfected cells, the protein level of nestin and ID-1 reduced greatly to an undetectable level, as expected, while in the TIMAP-overexpressing cell samples, although reduced, an apparent amount of nestin and ID-1 protein was however detected. These results clearly indicate that differentiation was delayed or incomplete in TIMAP-overexpressing cells.

### 2.4. TIMAP Translocate into the Nucleus of SH-SY5Y Cells upon Differentiation

**Figure 5:**
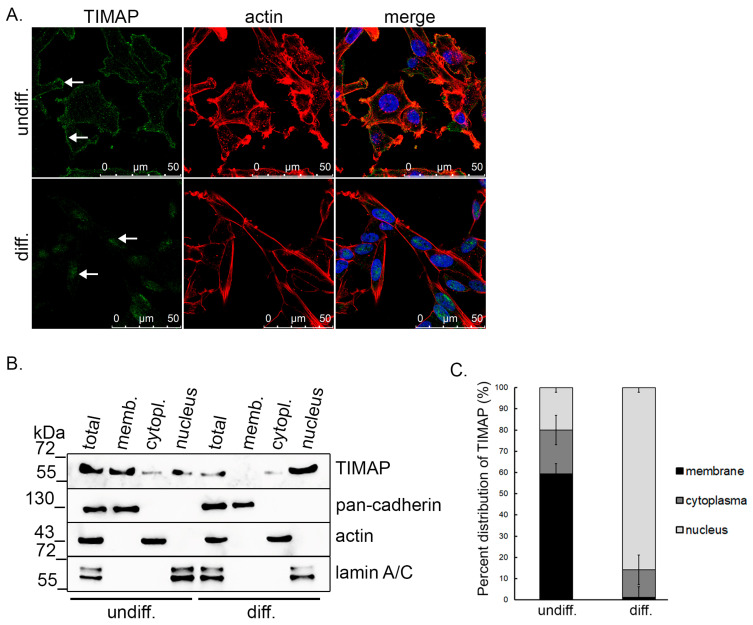
Change in the subcellular localization of TIMAP during differentiation. (**A**) Immunofluorescence staining of undifferentiated and differentiated (D6) cells was performed using anti-TIMAP (green) antibody. Actin filaments were observed utilizing Texas Red-Phalloidin, while the cell nuclei were labeled with DAPI. Scale bars: 50 µm. White arrows indicate TIMAP in the nucleus. (**B**) Subcellular fractions of control or differentiated (Day 6) cells were analyzed using anti-TIMAP antibody and antibodies specific for membrane (pan-cadherin), cytoplasm (actin) and nucleus (lamin A/C). (**C**) Normalized expression of TIMAP was determined by quantification of Western blots, and percent distribution ± SE of TIMAP protein in subcellular fraction were determined (*n* = 3).

As we showed above, TIMAP protein level decreases during differentiation. Therefore, immunofluorescent staining of undifferentiated and differentiated cells was carried out to study TIMAP subcellular localization. In undifferentiated SH-SY5Y cells, TIMAP was located in the membrane area, whereas in differentiated cells, TIMAP was predominantly observed in the nuclei (Figure 5A). This localization change was also studied by testing subcellular fractionations of the undifferentiated and differentiated cells by Western blot analysis. TIMAP was present with a higher abundancy in the membrane fraction of undifferentiated cells compared to the cytoplasmic and nuclear fractions. In differentiated cells, despite the lower total amount of the protein, a more pronounced appearance was found in the nucleus (Figure 5B).

To learn about the possible role of TIMAP in the nucleus, we initiated further studies to explore its nuclear interactome. Nuclear fraction of undifferentiated SH-SY5Y cells was performed and subsequently used in GST–TIMAP pull-down assay. Potential interacting proteins were separated on SDS-PAGE, and MS/MS analysis was performed. Fifty-seven potential TIMAP interacting partners were identified (Table 1).

**Figure 6:**
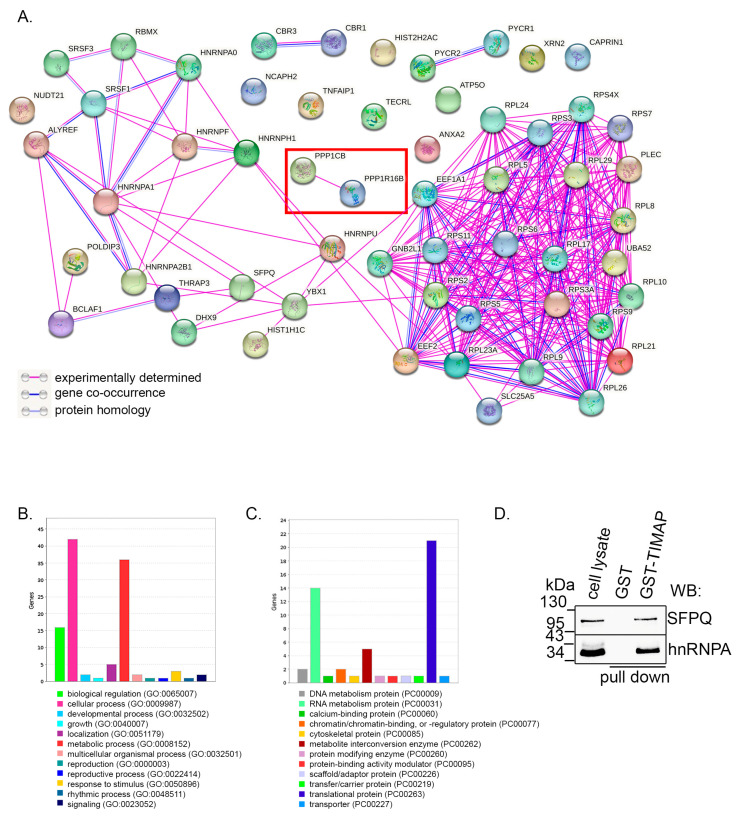
Analysis of nuclear protein interactions of TIMAP. (**A**) STRING network analysis of TIMAP binding proteins of SH-SY5Y nuclear fraction. Colors correspond to interactions according to the legend on the left. (**B**,**C**) Panther gene ontology (GO) term analysis of the nuclear interactome of TIMAP was performed (http://pantherdb.org (accessed on 9 September 2023)). Genes were categorized according to biological processes (**B**) or ontology was carried out by protein class categorization (**C**). (**D**) Purified nuclear fraction of SH-SY5Y cells were used in GST pull-down assay. Bound proteins were eluted and tested for SFPQ and hnRNPA1 by Western blot.

Figure 6A shows the STRING network analysis (https://string-db.org/; accessed on 9 September 2023) of the found proteins integrating the known and predicted associations in humans. Annexin A2 (ANXA2), eEF1A1 (eukaryotic elongation factor 1A) and RACK1 (GNBL21) interaction with TIMAP has already been reported in BPAEC cells [28,29,30]. To classify the proteins by their biological process (Figure 6B) and by their protein class (Figure 6C), panther gene ontology (GO) term analysis was performed (http://pantherdb.org; accessed on 9 September 2023). The majority of the identified proteins play a role in cellular and metabolic processes and belong to the translational and RNA metabolism protein classes. We also analyzed the direct interaction of two of the identified proteins, namely SFPQ and hnRNPA1. Figure 6D shows that both proteins are detectable in the pull-down samples.

## 3. Discussion

Neuronal differentiation involves intricate regulation by numerous signaling pathways. The complexity relies on gene expression up/downregulation; furthermore, the regulation is also influenced by posttranslational modifications of several proteins, such as reversible phosphorylation. Many recent studies have shown the involvement of protein phosphorylation and dephosphorylation not only in neuronal differentiation but also in many neurodegenerative disorders such as Parkinson’s or Alzheimer’s disease [31,32,33]. These findings presenting the importance of protein phosphatases were expected, as the brain expresses the highest levels of protein kinases and phosphatases of all mammalian tissues [34].

Protein phosphatases achieve their specificity through their regulatory subunits. TIMAP is considered as an endothelial-cell-predominant regulatory subunit of PP1, a Ser/Thr-specific protein phosphatase, and it belongs to the MYPT family [14]. In addition to the high expression level of TIMAP in endothelial cells, a reasonable amount was found in neuronal cells as well [14]. This piqued our interest to study the function of TIMAP in neuronal cells. The limited literature on the topic made the research challenging, but the present study focusing on TIMAP is just the first step to uncovering new protein phosphatase-1-based signaling mechanisms in neuronal cells.

The usage of B35 (rat neuroblastoma), PC12 (harvested from a pheochromocytoma rat adrenal medulla) or Neuro-2A (mouse neuroblast) cells is very common in neuronal signaling studies, but gene expression differences and signaling pathways alterations exist between these non-human cells vs. human cells [35,36,37]. Therefore, we used SH-SY5Y human neuroblastoma cell line that is originated from the SK-N-SH parental cell line obtained in 1973 through a multitude of subcloning [38].

In the present study, we have characterized a novel signaling pathway of neuronal differentiation involving TIMAP. We showed that SH-SY5Y cells express both TIMAP and PP1cδ, and the presence of the endogenous TIMAP–PP1c complex was also proved. The expression of PP1c isoforms in the brain was shown earlier, having the highest expression for PP1cα and PP1cγ, but all isoforms are considered as abundant [34]. Although TIMAP can bind all three isoforms of PP1c in vitro, it specifically interacts with the PP1cδ isoform in cells [39].

A multitude of approaches have been used for differentiation of SH-SY5Y cells including the standalone use or combination of serum deprivation, all-*trans* retinoic acid (RA) [40], dibutyryl cyclic AMP (dbcAMP) [41], brain-derived neurotropic factor (BDNF) [42], vanadate [43], nerve growth factor [44], estradiol (17-beta-estradiol), TPA (12-o-tetradecanoyl-pherbol-13-acetate) [45], cholesterol [46] and 1,25-dihydroxycholecalciferol (D3) [47]. With specific use of these differentiation agents, it is possible to produce a mature neuron-like cell population of dopaminergic, cholinergic or adrenergic neuronal phenotypes. Although many agents are in use to induce differentiation of SH-SY5Y cells, the effects of them on signaling pathways are less known. In contrast, retinoic acid, a vitamin A derivative, has well-documented effects on neuronal differentiation [48]. It is often used in 10 µM concentration through a differentiation period ranging from 5 to 21 days [25]. Retinoic acid signaling is mediated by two receptor types: the RXRs and the RARs, members of the nuclear hormone receptors acting by regulation of the transcriptional activity of their target genes [48].

All these advantages led us to choose the simplest protocol for SH-SY5Y differentiation using ATRA with the shortest incubation time to study the role of TIMAP protein in neuronal differentiation. Successful differentiation was confirmed by analyzing morphological changes as well as by the detection of expression of key differentiation markers. Interestingly, differentiated cells had lower *ppp1r16b* (TIMAP) mRNA level and, eventually, decreased TIMAP protein level. However, PP1c levels were not affected due to differentiation. In endothelial cells, TIMAP competes with MYPT1 for PP1c, and its overexpression reduces MYPT1 protein level [49]. MYPT1 expression in neuronal cells has been reported earlier [50]. Therefore, the expression of MYPT1 and MYPT3, members of the MYPT family that are largely homologous of TIMAP, was tested. We found that neither MYPT1 nor MYPT3 is up/downregulated upon differentiation; therefore, we concluded that during the differentiation of SH-SY5Y cells, the lower level of TIMAP is not compensated by an increased expression of other MYPT family members. Furthermore, our results demonstrated that overexpression of TIMAP in SH-SY5Y cells delayed neuronal differentiation. Not only nestin and ID-1 were detectable by Western blot in TIMAP-overexpressed and ATRA-treated cells on Day 6, but the cell morphology was resembling that of the immature phenotype. TIMAP-overexpressing cells had a significantly larger and rounder nucleus and their cytoplasm was smaller and shorter. This phenotype is more similar to the undifferentiated cells as differentiation results in an elongated cell morphology.

TIMAP was present mostly in the plasma membrane of SH-SY5Y, shown by cell fractionation and immunofluorescent staining. TIMAP has a C-terminal prenylation motif (CAAX-box) that allows its membrane localization [14]. In endothelial cells, an adaptor protein, RACK1, binds farnesyl transferase and TIMAP simultaneously and ensures C-terminal modification of TIMAP that directs the protein into the plasma membrane [28]. It was shown that the TIMAP–PP1c complex has several substrates in endothelial cell membrane, like ECE-1, ERM, merlin proteins (reviewed in [13]). Here, we showed the interaction of TIMAP–PP1c with RACK1 and ERM in SH-SY5Y cells as well.

Upon differentiation, we found that TIMAP translocated to the nuclear region of cells. TIMAP has an N-terminal bipartite nuclear localization signal that explains its nuclear appearance. The presence of TIMAP in the nucleus was detected earlier in pulmonary endothelial cells [12]. Yet, this is the first signaling pathway that was identified resulting in the translocation of TIMAP into the nucleus of cells. As TIMAP seems to inhibit differentiation, the nuclear interactome of TIMAP was analyzed in undifferentiated cells. With the possibility in mind that the decreased total protein levels may effect endogenous interaction detection, we carried out pull-down assay using recombinant TIMAP and the nuclear fraction of the undifferentiated SH-SY5Y cells. Although GST pull-down assay has limitations compared to endogenous IP (such as lack of posttranslational modifications of TIMAP), the nuclear interactome of TIMAP revealed numerous new potential binding partners of TIMAP besides the ones that were identified earlier in endothelial cells, namely ANXA2, eEF1A1 and RACK1 [28,29,30]. We showed before that eEF1A1 is involved in the nuclear trafficking of TIMAP, but it is not exclusively responsible for its nuclear export. However, eEF1A1 phosphorylated by ROCK seemed to be the substrate of the TIMAP–PP1c complex [30]. Several publications showed a decreased expression of eEF1A1 in Parkinson’s or Alzheimer’s disease patients [51,52,53]. The interaction of TIMAP with PP1cδ (PPP1CB) in the nucleus implies that the TIMAP–PP1c complex can have substrates in the nucleus. The nuclear interactome of TIMAP showed that its interacting partners are predominantly proteins involved in translation and RNA metabolism. Dysregulation of translation, phosphorylation of translational proteins can lead to serious conditions [54]. As there are several proteins that are found to form larger complexes from the identified partners, further verification of direct interaction with TIMAP should be conducted with specific assays. From the interactome, we chose heterogeneous nuclear ribonucleoprotein A1 (hnRNPA1) and splicing factor proline- and glutamine-rich (SFPQ) proteins to verify their interaction with TIMAP–PP1c. HnRNPA1 seems to be a hub protein among the interaction partners. It is known to be involved in pre-mRNA splicing, mRNA transport, RNA transcription and protein translation, and several studies showed that it contributes to neurodegenerative diseases too [55]. HnRNPA1 undergoes many posttranslational modifications including phosphorylation on Ser/Thr side chains [56,57,58,59]. Although the kinases responsible were identified, no phosphatases were described which ensure reversibility. One may hypothesize that TIMAP–PP1c could be responsible for dephosphorylation of (some of) these side chains; however, this requires further investigation. The other examined interacting partner is SFPQ, an RNA-binding protein involved in neuronal function and neurodegeneration [60]. SFPQ is a multifunctional protein as it interacts with several proteins regulating paraspeckle formation, alternative splicing or DNA damage repair, and axon growth [61,62,63]. It was shown in T cells that SFPQ is phosphorylated on Thr687 by GSK3 [64], offering another future path for our research.

In conclusion, the TIMAP–PP1c complex was shown to be present in neuronal cells, and TIMAP appears to be an inhibitor of neuronal differentiation. The nuclear interactome of TIMAP revealed several new potential protein binding partners, but the detailed signaling pathways remain to be investigated. These binding partners can be novel substrates of TIMAP–PP1c and further demonstrate the possible role of TIMAP as a transcriptional regulator.

## 4. Materials and Methods

### 4.1. Cell Cultures and Cell Differentiation

Human SH-SY5Y (European Tissue Culture) cells were maintained in DMEM containing 10% heat inactivated FBS, 2 mM of L-glutamine and 4.5 g/L of glucose at 37 °C in a 5% CO_2_ incubator. Next, 10 μM of all-*trans* retinoic acid (ATRA) in 1% FBS containing DMEM was used for 6 days for SH-SY5Y cell differentiation. Medium was replenished every 48 h [21,65]. Differentiation was monitored via microscopic assessment of morphological changes of neurite outgrowth or by collecting the samples for Western blot or qPCR. BPAEC cells were used and maintained as described earlier [28].

### 4.2. Bacterial Protein Expression and GST Pull-Down Assay, LC-MS/MS Analysis

GST or GST–TIMAP proteins were formed and purified as described in [28]. SH-SY5Y cells were lysed in 800 μL 50 mM of Tris-HCl (pH 7.5), 0.2% 2-mercaptoethanol, 0.1% Tween 20 containing lysis buffer in the presence of protease inhibitors. Pulldown was performed as described in [28].

Mass Spectrometry was performed as described in [28].

### 4.3. SDS-PAGE and Western Blotting

Denatured protein samples (30–50 μg total protein) were separated on SDS polyacrylamide gels (10–12%), and Western blot was carried out as described in [29]. In each individual experiment, equal amounts of protein were loaded per well. Primary and secondary antibodies employed are listed in Table 2.

### 4.4. RNA Isolation, cDNA Synthesis and qPCR

SH-SY5Y cells were utilized to extract total RNA using a GeneJET RNA purification kit (Thermo Scientific, Waltham, MA, USA). Then, 2 µg of total RNA was employed for cDNA synthesis using Maxima Reverse Transcriptase™ (Thermo Scientific) and oligo-d(T)_16_ primer (Promega, Madison, WI, USA).

Real-time PCR analysis was performed with a LightCycler 480 Thermocycler (Roche, Basel, Switzerland, Europe) and Maxima SYBR Green qPCR Master Mix (Thermo Scientific) according to the manufacturer’s protocol. GAPDH was used to normalized threshold values (C_t_ values). Fold expression levels were calculated by 2^−ΔΔCt^ values as in [66]. Applied primers are listed in Table 3; synthesized by Integrated DNA Technologies (Leuven, Belgium, Europe).

### 4.5. Immunoprecipitation, Immunofluorescence and Microscopy

Immunoprecipitation was performed as described in [28]. Immunofluorescent staining and microscopy were performed as described in [29], TIMAP antibody was used in 1:100 dilution, and Texas-Red Phalloidin (Invitrogen, Waltham, MA, USA) was used in 1:300 dilution.

### 4.6. Transfection

In order to overexpress TIMAP, SH-SY5Y cells were transfected with pEGFP-C1 TIMAP wild-type plasmid (coding for human TIMAP protein), constructed earlier [28] using Lipofectamine 3000 reagent according to the manufacturer’s protocol.

### 4.7. Subcellular Fractionation

Subcellular fractions of SH-SY5Y cells were formed as described in [28].

### 4.8. High-Content Analysis

SH-SY5Y cells were plated into CellCarrier Ultra 96-well microplates, and the indicated treatments were carried out. Subsequently, images were captured using an Opera Phenix high-content screening system (Perkin Elmer, Waltham, MA, USA) equipped with a 10× air objective, and the images were then analyzed using the integrated Harmony software (version 4.8, Perkin Elmer) [67].

### 4.9. Statistical Analysis

The GraphPad Prism program (version 8.0.1) was used for statistical analysis. Significances and applied statistical tests are indicated at each experiment. Image J software (version 1.54) was used for densitometry.

## Figures and Tables

**Table 1:** GST–TIMAP binding proteins of SH-SY5Y nuclear fraction.

Protein ID (UniProt)	Protein Name	Nr. of Peptide	Gene Name
Q15149	Plectin	290	*PLEC*
Q08211	ATP-dependent RNA helicase A	61	*DHX9*
Q9H0D6	5-3 exoribonuclease 2	47	*XRN2*
P13639	Elongation factor 2	45	*EEF2*
Q9Y2W1	Thyroid-hormone-receptor-associated protein 3	42	*THRAP3*
Q00839	Heterogeneous nuclear ribonucleoprotein U	37	*HNRNPU*
Q9NYF8	Bcl-2-associated transcription factor 1	35	*BCLAF1*
P23246	Splicing factor, proline- and glutamine-rich	30	*SFPQ*
Q6IBW4	Condensin-2 complex subunit H2	26	*NCAPH2*
P38159	RNA-binding motif protein	25	*RBMX*
Q96T49	Protein phosphatase 1 regulatory inhibitor subunit 16B	24	*PPP1R16B*
Q9BY77	Polymerase delta-interacting protein 3	24	*POLDIP3*
P07355	Annexin A2	23	*ANXA2*
Q5VTE0	Elongation factor 1-alpha 1	21	*EEF1A1*
Q13829	BTB/POZ domain-containing adapter for CUL3-mediated RhoA degradation protein 2	21	*TNFAIP1*
Q14444	Caprin-1	21	*CAPRIN1*
P22626	Heterogeneous nuclear ribonucleoproteins A2/B1	20	*HNRNPA2B1*
P63244	Guanine nucleotide-binding protein subunit beta-2-like 1	20	*GNB2L1*
P05141	ADP/ATP translocase 2	19	*SLC25A5*
P23396	40S ribosomal protein S3	19	*RPS3*
P09651	Heterogeneous nuclear ribonucleoprotein A1	18	*HNRNPA1*
P15880	40S ribosomal protein S2	18	*RPS2*
P31943	Heterogeneous nuclear ribonucleoprotein H	18	*HNRNPH1*
P16152	Carbonyl reductase [NADPH] 1	17	*CBR1*
P61247	40S ribosomal protein S3a	17	*RPS3A*
P62140	Serine/threonine-protein phosphatase PP1-beta catalytic subunit	17	*PPP1CB*
O75828	Carbonyl reductase [NADPH] 3	16	*CBR3*
P62701	40S ribosomal protein S4, X isoform	16	*RPS4X*
Q96C36	Pyrroline-5-carboxylate reductase 2	16	*PYCR2*
P32322	Pyrroline-5-carboxylate reductase 1, mitochondrial	15	*PYCR1*
P52597	Heterogeneous nuclear ribonucleoprotein F	15	*HNRNPF*
O43809	Cleavage and polyadenylation specificity factor subunit 5	14	*NUDT21*
P67809	Nuclease-sensitive element-binding protein 1	13	*YBX1*
Q07955	Serine/arginine-rich splicing factor 1	13	*SRSF1*
Q13151	Heterogeneous nuclear ribonucleoprotein A0	13	*HNRNPA0*
Q86V81	THO complex subunit 4	13	*ALYREF*
P48047	ATP synthase subunit O, mitochondrial	12	*ATP5O*
Q5HYJ1	Trans-2,3-enoyl-CoA reductase-like	12	*TECRL*
P46777	60S ribosomal protein L5	11	*RPL5*
P46781	40S ribosomal protein S9	11	*RPS9*
P46782	40S ribosomal protein S5	10	*RPS5*
P62081	40S ribosomal protein S7	10	*RPS7*
P62280	40S ribosomal protein S11	10	*RPS11*
P62917	60S ribosomal protein L8	10	*RPL8*
P16403	Histone H1.2	9	*HIST1H1C*
P18621	60S ribosomal protein L17	9	*RPL17*
P32969	60S ribosomal protein L9	9	*RPL9*
P27635	60S ribosomal protein L10	8	*RPL10*
P62753	40S ribosomal protein S6	8	*RPS6*
P83731	60S ribosomal protein L24	8	*RPL24*
P84103	Serine/arginine-rich splicing factor 3	7	*SRSF3*
P62987	Ubiquitin-60S ribosomal protein L40	6	*UBA52*
P46778	60S ribosomal protein L21	6	*RPL21*
P61254	60S ribosomal protein L26;60S ribosomal protein L26-like 1	6	*RPL26*
P62750	60S ribosomal protein L23a	6	*RPL23A*
Q16777	Histone H2A type 2-C	5	*HIST2H2AC*
P47914	60S ribosomal protein L29	4	*RPL29*

**Table 2:** Primary and secondary antibodies utilized in Western blot (WB).

Antibody	Dilution	Vendor (cat. #)
TIMAP	1:1000	ABGENT (San Diego, CA, USA)(AP17590c)
PP1cδ	1:1000	Millipore (Burlington, MA, USA) (#07-270)
PP1cα	1:1000	R&D Systems (Minneapolis, MN, USA) (MAB6105)
actin	1:1000	Sigma (St. Louis, MO, USA) (A5060)
ERM	1:1000	Cell Signaling Technologies (Danvers, MA, USA) (#3142)
merlin	1:1000	Cell Signaling Technologies (#6995)
RACK1	1:1000	BD Transduction Laboratories (Franklin Lakes, NJ, USA) (#610177)
GFP	1:1000	Millipore (#AB10145; #MAB1083)
Nestin	1:2000	Cell Signaling Technologies (#4760)
ID-1	1:500	Novus Biologicals (Littleton, CO, USA) (#H00003397-M02)
lamin A/C	1:2000	Santa Cruz (Dallas, TX, USA) (sc-20681)
pan-cadherin	1:1000	Cell Signaling Technologies (#4068)
HRP-linked anti-rabbit IgG	1:5000	Cell Signaling Technologies (#7074)
HRP-linked anti-mouse IgG	1:5000	Cell Signaling Technologies (#7076)

**Table 3:** Primer pair nucleotide sequences utilized in real-time PCR experiments.

Gene Name	Primer 1	Primer 2
*GAPDH* (glyceraldehyde-3-phosphate dehydrogenase)	5′-GTC TCC TCT GAC TTC AAC AGC G-3′	5′-ACC ACC CTG TTG CTG TAG CCA A-3′
*NES* (nestin)	5′-TCA AGA TGT CCC TCA GCC TGG A-3′	5′-AAG CTG AGG GAA GTC TTG GAG C-3′
*PCNA* (proliferating cell nuclear antigen)	5′-CAA GTA ATG TCG ATA AAG AGG AGG-3′	5′-GTG TCA CCG TTG AAG AGA GTG G-3′
*ID1* (Inhibitor of differentiation 1)	5′-GTT GGA GCT GAA CTC GGA ATC C-3′	5′-ACA CAA GAT GCG ATC GTC CGC A-3′
*TUBB3* (β3-tubulin)	5′-TCA GCG TCT ACT ACA ACG AGG C-3′	5′-GCC TGA AGA GAT GTC CAA AGG C-3′
*MAP2* (microtubule-associated protein 2)	5′-AGG CTG TAG CAG TCC TGA AAG G-3′	5′-CTT CCT CCA CTG TGA CAG TCT G-3′
*NeuN* (RBFOX3)	5′-TAC GCA GCC TAC AGA TAC GCT C-3′	5′-TGG TTC CAA TGC TGT AGG TCG C-3′
*SYP* (synaptophysin)	5′-TCG GCT TTG TGA AGG TGC TGC A-3′	5′-TCA CTC TCG GTC TTG TTG GCA C-3′
*ACTB* (b-actin)	5′-CAC CAT TGG CAA TGA GCG GTT C-3′	5′-AGG TCT TTG CGG ATG TCC ACG T-3′
*PPP1CA* (PP1c α)	5′-GCT GCT GGC CTA TAA GAT CAA-3′	5′-GTC TCT TGC ACT CAT CGT AGA A-3′
*PPP1CB* (PP1c δ)	5′-CAG CCA TTG TGG ATG AGA AGA-3′	5′-AGG GAC ATC AGT AGG TCT CAT AA-3′
*PPP1R16B* (TIMAP)	5′-TGG AGC TAG TCT CAG TGC AAG G-3′	5′-CTC AAG GAT GAC TTG TGC CTC AG-3′
*PPP1R12A* (MYPT1)	5′-CAG GTC AAG CAA CAC CTA CA-3′	5′-GCC CGT CTT TCT AAG TCC TAA C-3′
*PPP1R16A* (MYPT3)	5′-TCA ATG CCT GTG ACA GTG AG-3′	5′-ATG TTC CCG TCG GTG TTG-3′

## Data Availability

The data that support the findings of this study are available from the corresponding author upon reasonable request.

## Supplementary Materials

The following supporting information can be downloaded at: https://www.mdpi.com/article/10.3390/ijms242417360/s1.

## References

[1] Barford D. (1995). Protein phosphatases. Curr. Opin. Struct. Biol..

[2] Ariño J., Velázquez D., Casamayor A. (2019). Ser/Thr protein phosphatases in fungi: Structure, regulation and function. Microb. Cell.

[3] Cohen P.T., Brewis N.D., Hughes V., Mann D.J. (1990). Protein serine/threonine phosphatases; an expanding family. FEBS Lett..

[4] Shi Y. (2009). Serine/Threonine Phosphatases: Mechanism through Structure. Cell.

[5] Bollen M., Peti W., Ragusa M.J., Beullens M. (2010). The extended PP1 toolkit: Designed to create specificity. Trends Biochem. Sci..

[6] Cannon J.F. (2010). Function of protein phosphatase-1, Glc7, in Saccharomyces cerevisiae. Adv. Appl. Microbiol..

[7] Peti W., Nairn A.C., Page R. (2013). Structural basis for protein phosphatase 1 regulation and specificity. FEBS J..

[8] Korrodi-Gregorio L., Esteves S.L., Fardilha M. (2014). Protein phosphatase 1 catalytic isoforms: Specificity toward interacting proteins. Transl. Res..

[9] Ceulemans H., Bollen M. (2004). Functional diversity of protein phosphatase-1, a cellular economizer and reset button. Physiol. Rev..

[10] Gibbons J.A., Kozubowski L., Tatchell K., Shenolikar S. (2007). Expression of human protein phosphatase-1 in Saccharomyces cerevisiae highlights the role of phosphatase isoforms in regulating eukaryotic functions. J. Biol. Chem..

[11] Verbinnen I., Ferreira M., Bollen M. (2017). Biogenesis and activity regulation of protein phosphatase 1. Biochem. Soc. Trans..

[12] Csortos C., Czikora I., Bogatcheva N.V., Adyshev D.M., Poirier C., Olah G., Verin A.D. (2008). TIMAP is a positive regulator of pulmonary endothelial barrier function. Am. J. Physiol. Lung Cell. Mol. Physiol..

[13] Boratko A., Csortos C. (2017). TIMAP, the versatile protein phosphatase 1 regulator in endothelial cells. IUBMB Life.

[14] Cao W., Mattagajasingh S.N., Xu H., Kim K., Fierlbeck W., Deng J., Lowenstein C.J., Ballermann B.J. (2002). TIMAP, a novel CAAX box protein regulated by TGF-beta1 and expressed in endothelial cells. Am. J. Physiol. Cell Physiol..

[15] Czikora I., Kim K.M., Kasa A., Becsi B., Verin A.D., Gergely P., Erdodi F., Csortos C. (2011). Characterization of the effect of TIMAP phosphorylation on its interaction with protein phosphatase 1. Biochimie.

[16] Boratko A., Csortos C. (2017). PKC mediated phosphorylation of TIMAP regulates PP1c activity and endothelial barrier function. Biochim. Biophys. Acta Mol. Cell Res..

[17] Schulz A., Zoch A., Morrison H. (2014). A neuronal function of the tumor suppressor protein merlin. Acta Neuropathol. Commun..

[18] Magdaleno S., Northcutt G.M., Curran T., Kurschner C. (2002). mPPP1R16B is a novel mouse protein phosphatase 1 targeting subunit whose mRNA is located in cell bodies and dendrites of neurons in four distinct regions of the brain. Brain Res. Gene Expr. Patterns.

[19] Alivand M.R., Najafi S., Esmaeili S., Rahmanpour D., Zhaleh H., Rahmati Y. (2021). Integrative analysis of DNA methylation and gene expression profiles to identify biomarkers of glioblastoma. Cancer Genet..

[20] Bell M., Zempel H. (2021). SH-SY5Y-derived neurons: A human neuronal model system for investigating TAU sorting and neuronal subtype-specific TAU vulnerability. Rev. Neurosci..

[21] Xicoy H., Wieringa B., Martens G.J. (2017). The SH-SY5Y cell line in Parkinson’s disease research: A systematic review. Mol. Neurodegener..

[22] Strother L., Miles G.B., Holiday A.R., Cheng Y., Doherty G.H. (2021). Long-term culture of SH-SY5Y neuroblastoma cells in the absence of neurotrophins: A novel model of neuronal ageing. J. Neurosci. Methods.

[23] De Conto V., Cheung V., Maubon G., Souguir Z., Maubon N., Vandenhaute E., Berezowski V. (2021). In vitro differentiation modifies the neurotoxic response of SH-SY5Y cells. Toxicol. In Vitro.

[24] Christensen J., Steain M., Slobedman B., Abendroth A. (2011). Differentiated neuroblastoma cells provide a highly efficient model for studies of productive varicella-zoster virus infection of neuronal cells. J. Virol..

[25] Shipley M.M., Mangold C.A., Szpara M.L. (2016). Differentiation of the SH-SY5Y Human Neuroblastoma Cell Line. J. Vis. Exp..

[26] Lopes F.M., Schroder R., da Frota M.L., Zanotto-Filho A., Muller C.B., Pires A.S., Meurer R.T., Colpo G.D., Gelain D.P., Kapczinski F. (2010). Comparison between proliferative and neuron-like SH-SY5Y cells as an in vitro model for Parkinson disease studies. Brain Res..

[27] Campos Cogo S., Gradowski Farias da Costa do Nascimento T., de Almeida Brehm Pinhatti F., de Franca Junior N., Santos Rodrigues B., Cavalli L.R., Elifio-Esposito S. (2020). An overview of neuroblastoma cell lineage phenotypes and in vitro models. Exp. Biol. Med..

[28] Boratko A., Gergely P., Csortos C. (2013). RACK1 is involved in endothelial barrier regulation via its two novel interacting partners. Cell Commun. Signal..

[29] Kiraly N., Thalwieser Z., Fonodi M., Csortos C., Boratko A. (2021). Dephosphorylation of annexin A2 by protein phosphatase 1 regulates endothelial cell barrier. IUBMB Life.

[30] Boratko A., Peter M., Thalwieser Z., Kovacs E., Csortos C. (2015). Elongation factor-1A1 is a novel substrate of the protein phosphatase 1-TIMAP complex. Int. J. Biochem. Cell Biol..

[31] Khakha N., Khan H., Kaur A., Singh T.G. (2023). Therapeutic implications of phosphorylation- and dephosphorylation-dependent factors of cAMP-response element-binding protein (CREB) in neurodegeneration. Pharmacol. Rep..

[32] Brembati V., Faustini G., Longhena F., Outeiro T.F., Bellucci A. (2023). Changes in alpha-Synuclein Posttranslational Modifications in an AAV-Based Mouse Model of Parkinson’s Disease. Int. J. Mol. Sci..

[33] Li Z., Yin B., Zhang S., Lan Z., Zhang L. (2023). Targeting protein kinases for the treatment of Alzheimer’s disease: Recent progress and future perspectives. Eur. J. Med. Chem..

[34] Da Cruz e Silva E.F., Fox C.A., Ouimet C.C., Gustafson E., Watson S.J., Greengard P. (1995). Differential expression of protein phosphatase 1 isoforms in mammalian brain. J. Neurosci..

[35] Otey C.A., Boukhelifa M., Maness P. (2003). B35 neuroblastoma cells: An easily transfected, cultured cell model of central nervous system neurons. Methods Cell Biol..

[36] Shafer T.J., Atchison W.D. (1991). Transmitter, ion channel and receptor properties of pheochromocytoma (PC12) cells: A model for neurotoxicological studies. Neurotoxicology.

[37] LePage K.T., Dickey R.W., Gerwick W.H., Jester E.L., Murray T.F. (2005). On the use of neuro-2a neuroblastoma cells versus intact neurons in primary culture for neurotoxicity studies. Crit. Rev. Neurobiol..

[38] Biedler J.L., Helson L., Spengler B.A. (1973). Morphology and growth, tumorigenicity, and cytogenetics of human neuroblastoma cells in continuous culture. Cancer Res..

[39] Shopik M.J., Li L., Luu H.A., Obeidat M., Holmes C.F., Ballermann B.J. (2013). Multi-directional function of the protein phosphatase 1 regulatory subunit TIMAP. Biochem. Biophys. Res. Commun..

[40] Singh J., Kaur G. (2007). Transcriptional regulation of polysialylated neural cell adhesion molecule expression by NMDA receptor activation in retinoic acid-differentiated SH-SY5Y neuroblastoma cultures. Brain Res..

[41] Kume T., Kawato Y., Osakada F., Izumi Y., Katsuki H., Nakagawa T., Kaneko S., Niidome T., Takada-Takatori Y., Akaike A. (2008). Dibutyryl cyclic AMP induces differentiation of human neuroblastoma SH-SY5Y cells into a noradrenergic phenotype. Neurosci. Lett..

[42] Cernaianu G., Brandmaier P., Scholz G., Ackermann O.P., Alt R., Rothe K., Cross M., Witzigmann H., Trobs R.B. (2008). All-trans retinoic acid arrests neuroblastoma cells in a dormant state. Subsequent nerve growth factor/brain-derived neurotrophic factor treatment adds modest benefit. J. Pediatr. Surg..

[43] Rogers M.V., Buensuceso C., Montague F., Mahadevan L. (1994). Vanadate stimulates differentiation and neurite outgrowth in rat pheochromocytoma PC12 cells and neurite extension in human neuroblastoma SH-SY5Y cells. Neuroscience.

[44] Oe T., Sasayama T., Nagashima T., Muramoto M., Yamazaki T., Morikawa N., Okitsu O., Nishimura S., Aoki T., Katayama Y. (2005). Differences in gene expression profile among SH-SY5Y neuroblastoma subclones with different neurite outgrowth responses to nerve growth factor. J. Neurochem..

[45] Pahlman S., Odelstad L., Larsson E., Grotte G., Nilsson K. (1981). Phenotypic changes of human neuroblastoma cells in culture induced by 12-O-tetradecanoyl-phorbol-13-acetate. Int. J. Cancer.

[46] Sarkanen J.R., Nykky J., Siikanen J., Selinummi J., Ylikomi T., Jalonen T.O. (2007). Cholesterol supports the retinoic acid-induced synaptic vesicle formation in differentiating human SH-SY5Y neuroblastoma cells. J. Neurochem..

[47] Agholme L., Lindstrom T., Kagedal K., Marcusson J., Hallbeck M. (2010). An in vitro model for neuroscience: Differentiation of SH-SY5Y cells into cells with morphological and biochemical characteristics of mature neurons. J. Alzheimers Dis..

[48] Clagett-Dame M., McNeill E.M., Muley P.D. (2006). Role of all-trans retinoic acid in neurite outgrowth and axonal elongation. J. Neurobiol..

[49] Wang X., Obeidat M., Li L., Pasarj P., Aburahess S., Holmes C.F.B., Ballermann B.J. (2019). TIMAP inhibits endothelial myosin light chain phosphatase by competing with MYPT1 for the catalytic protein phosphatase 1 subunit PP1cbeta. J. Biol. Chem..

[50] Lontay B., Serfozo Z., Gergely P., Ito M., Hartshorne D.J., Erdodi F. (2004). Localization of myosin phosphatase target subunit 1 in rat brain and in primary cultures of neuronal cells. J. Comp. Neurol..

[51] Beckelman B.C., Day S., Zhou X., Donohue M., Gouras G.K., Klann E., Keene C.D., Ma T. (2016). Dysregulation of Elongation Factor 1A Expression is Correlated with Synaptic Plasticity Impairments in Alzheimer’s Disease. J. Alzheimers Dis..

[52] Beckelman B.C., Zhou X., Keene C.D., Ma T. (2016). Impaired Eukaryotic Elongation Factor 1A Expression in Alzheimer’s Disease. Neurodegener. Dis..

[53] Garcia-Esparcia P., Hernandez-Ortega K., Koneti A., Gil L., Delgado-Morales R., Castano E., Carmona M., Ferrer I. (2015). Altered machinery of protein synthesis is region- and stage-dependent and is associated with alpha-synuclein oligomers in Parkinson’s disease. Acta Neuropathol. Commun..

[54] Kapur M., Monaghan C.E., Ackerman S.L. (2017). Regulation of mRNA Translation in Neurons-A Matter of Life and Death. Neuron.

[55] Clarke J.P., Thibault P.A., Salapa H.E., Levin M.C. (2021). A Comprehensive Analysis of the Role of hnRNP A1 Function and Dysfunction in the Pathogenesis of Neurodegenerative Disease. Front. Mol. Biosci..

[56] Roy R., Durie D., Li H., Liu B.Q., Skehel J.M., Mauri F., Cuorvo L.V., Barbareschi M., Guo L., Holcik M. (2014). hnRNPA1 couples nuclear export and translation of specific mRNAs downstream of FGF-2/S6K2 signalling. Nucleic Acids Res..

[57] Choi Y.H., Lim J.K., Jeong M.W., Kim K.T. (2012). HnRNP A1 phosphorylated by VRK1 stimulates telomerase and its binding to telomeric DNA sequence. Nucleic Acids Res..

[58] Koo J.H., Lee H.J., Kim W., Kim S.G. (2016). Endoplasmic Reticulum Stress in Hepatic Stellate Cells Promotes Liver Fibrosis via PERK-Mediated Degradation of HNRNPA1 and Up-regulation of SMAD2. Gastroenterology.

[59] Martin J., Masri J., Cloninger C., Holmes B., Artinian N., Funk A., Ruegg T., Anderson L., Bashir T., Bernath A. (2011). Phosphomimetic substitution of heterogeneous nuclear ribonucleoprotein A1 at serine 199 abolishes AKT-dependent internal ribosome entry site-transacting factor (ITAF) function via effects on strand annealing and results in mammalian target of rapamycin complex 1 (mTORC1) inhibitor sensitivity. J. Biol. Chem..

[60] Lim Y.W., James D., Huang J., Lee M. (2020). The Emerging Role of the RNA-Binding Protein SFPQ in Neuronal Function and Neurodegeneration. Int. J. Mol. Sci..

[61] Sasaki Y.T., Ideue T., Sano M., Mituyama T., Hirose T. (2009). MENepsilon/beta noncoding RNAs are essential for structural integrity of nuclear paraspeckles. Proc. Natl. Acad. Sci. USA.

[62] Ha K., Takeda Y., Dynan W.S. (2011). Sequences in PSF/SFPQ mediate radioresistance and recruitment of PSF/SFPQ-containing complexes to DNA damage sites in human cells. DNA Repair.

[63] Kim K.K., Kim Y.C., Adelstein R.S., Kawamoto S. (2011). Fox-3 and PSF interact to activate neural cell-specific alternative splicing. Nucleic Acids Res..

[64] Heyd F., Lynch K.W. (2010). Phosphorylation-dependent regulation of PSF by GSK3 controls CD45 alternative splicing. Mol. Cell.

[65] Ferguson R., Subramanian V. (2016). PA6 Stromal Cell Co-Culture Enhances SH-SY5Y and VSC4.1 Neuroblastoma Differentiation to Mature Phenotypes. PLoS ONE.

[66] Livak K.J., Schmittgen T.D. (2001). Analysis of relative gene expression data using real-time quantitative PCR and the 2(-Delta Delta C(T)) Method. Methods.

[67] Regdon Z., Demeny M.A., Kovacs K., Hajnady Z., Nagy-Penzes M., Bakondi E., Kiss A., Hegedus C., Virag L. (2021). High-content screening identifies inhibitors of oxidative stress-induced parthanatos: Cytoprotective and anti-inflammatory effects of ciclopirox. Br. J. Pharmacol..

