# Supplementary material for: TIMAP, a Regulatory Subunit of Protein Phosphatase 1, Inhibits In Vitro Neuronal Differentiation
Source: Int J Mol Sci. 2023 Dec 11;24(24):17360. doi: 10.3390/ijms242417360 (PMC10744335; doi:10.3390/ijms242417360)
Supplement: Supplementary file 1 [file ijms-24-17360-s001.zip › ijms-2703255-supplementary.pdf]

SFig 1.

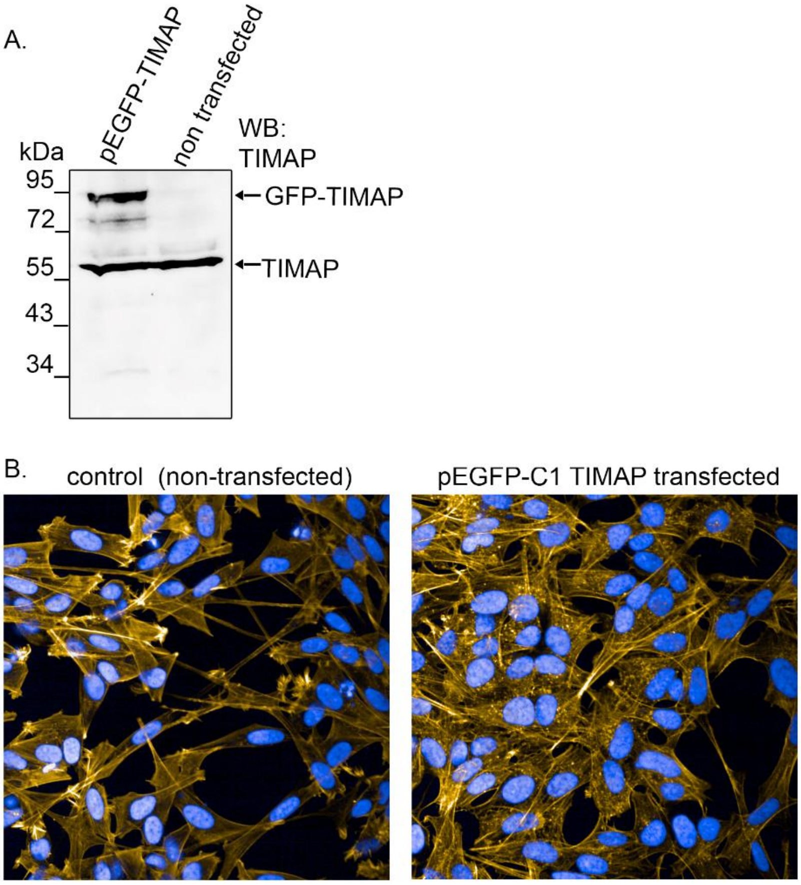

Figure S1. (**A**) Overexpression of TIMAP was tested using anti-TIMAP antibody in Western blot experiment. TIMAP antibody recognized endogenous and GFP-tagged TIMAP protein. (**B**) Differentiated control and pEGFP-C1 TIMAP-expressing cells were analyzed using Opera Phenix HCS. Actin filaments were stained with Texas Red Phalloidin, and nuclei were visualized by DAPI staining.
